# Effectiveness of distance learning strategies for continuing professional development (CPD) for rural allied health practitioners: a systematic review

**DOI:** 10.1186/s12909-017-0949-5

**Published:** 2017-07-12

**Authors:** Angela Berndt, Carolyn M. Murray, Kate Kennedy, Mandy J. Stanley, Susan Gilbert-Hunt

**Affiliations:** University of South Australia, School of Health Sciences, Adelaide, Australia

**Keywords:** Continuing professional development, Allied health professionals, Distance education, Rural health workforce, Education technology

## Abstract

**Background:**

Allied health professionals working in rural areas face unique challenges, often with limited access to resources. Accessing continuing professional development is one of those challenges and is related to retention of workforce. Effectiveness of distance learning strategies for continuing professional development in rural allied healthcare workers has not been evaluated.

**Methods:**

We searched 17 databases and the grey literature up to September 2016 following the PRISMA guidelines. Any primary studies were included that focussed on allied health and distance delivery regardless of education topic or study design. Two independent reviewers extracted data and critically appraised the selected studies.

**Results:**

The search returned 5257 results. With removal of duplicate references, we reviewed 3964 article titles and abstracts; *n* = 206 appeared potentially eligible and were scrutinised via full text screening; *n* = 14 were included. Studies were published between 1997 and 2016, were of varied methodological quality and were predominantly from Australia, USA and Canada with a focus on satisfaction of learners with the delivery method or on measures of educational outcomes. Technologies used to deliver distance education included video conference, teleconference, web based platforms and virtual reality. Early papers tended to focus more on the technology characteristics than educational outcomes. Some studies compared technology based delivery to face to face modes and found satisfaction and learning outcomes to be on par. Only three studies reported on practice change following the educational intervention and, despite a suggestion there is a link between the constructs, none measured the relationship between access to continuing professional development and workforce retention.

**Conclusion:**

Technology based options of delivery have a high utility, however the complex inter-relatedness of time, use, travel, location, costs, interactivity, learning outcomes and educational design suggest a need for more sophisticated consideration by educational providers.

**Trial registration:**

Registration with PROSPERO 30 June 2016: CRD42016041588.

## Background

The context of working in rural allied health is unique. Rural allied health practitioners (AHP) are confronted with a broad range of challenges in daily practice requiring an extensive general skill-base to cope with the diversity and demands of clients, often in an environment where resources are scarce and there are minimal support structures [1–6]. In addition, delivery methods for health services are constantly changing, requiring AHP to be adaptable and responsive to new demands placed upon them. A recent example of changes to health services is the introduction of the National Disability Insurance Scheme (NDIS) in Australia. The NDIS is expected to generate a responsive, person centred service which enables people with disability to choose when and how they receive support from health professionals [7]. In the new scheme, rural AHP may be required to assess individual’s needs that previously may not have been part of their practice experience, leading to an even greater need for training and continuing professional development (CPD) opportunities in this group.

CPD is offered by employers or other providers and taken up by AHP to enhance knowledge, skills competence and performance in order to improve patient and client outcomes [8]. CPD is typically offered via educational meetings that are either interactive or didactic and usually utilise printed educational materials or other resources as a component of the intervention [8]. Educational meetings are defined as conferences, lectures, workshops, seminars, symposia and courses with evidence suggesting that mixed interactive and didactic education is more effective than either alone [8]. Lack of access to CPD is known to be problematic for rural AHP [9, 10]. In particular, rural AHP cite additional costs of travel to attend CPD [11], expensive registrations [12] and not being provided with a car or time to travel [9].

Attracting and then retaining a rural AHP work force is itself a challenge [11, 13] with reasons cited including the requirement to be generalist AHP and the need to be both administrators and health service providers [9]. Different methods have been considered for provision of CPD to rural AHP [14] to support recruitment and retention [15]. In particular, transdisciplinary and interdisciplinary approaches have been promoted as there may only be one person from each discipline in rural centres and these approaches allow the exchange of ideas, skills and information amongst the team [16, 17]. CPD provided by distance education is another response to overcome the barriers associated with travel distance and cost.

The availability of distance education, subsidised CPD and use of technology to deliver education or training to rural Australia was thought to allow cost-effective and equitable access to CPD for rural AHP [5, 18, 19]. However, other research suggested that methods utilising technologies for delivery of CPD, while helpful in enabling AHP to learn locally, may not fully meet their needs [4] as they needed ‘time out’ to learn [10] and it could not replace face-to-face contact [11]. Therefore, while email, video-conferencing and internet-based programs have some place in CPD for AHP, they may not allow full interaction and collaborative learning between the educator and AHP. These limitations of distance education may account for limited uptake in the past [4] and current variability amongst rural AHP [20].

Due to the uniqueness of the experiences and the demands placed on rural AHP, more needs to be known about what technological and learning strategies are most beneficial for supporting the CPD of AHP working in rural settings. For this reason, we undertook a systematic review with the aim of evaluating the effectiveness of distance learning strategies to provide CPD to rural AHP. There are two aspects to this review question; what distance learning strategies are currently used to provide CPD for rural AHP and; how effective are these strategies in improving rural AHP outcomes. Outcomes of interest were practitioner knowledge change; practitioner confidence change; practice change; and practitioner satisfaction with the CPD distance learning model used.

## Methods

The systematic review of the effectiveness of distance learning strategies for rural AHP followed the PRISMA statement guidelines [21], and the search protocol was prospectively registered with PROSPERO (registration number CRD42016041588, 30 June 2016). The following databases were searched: Informit health collection; Medline; AMED; Academic Search Premier; Australian and New Zealand Reference Centre; CINAHL; Health Source: Nursing/academic edition; Cochrane library; Scopus; Web of Science; Google Scholar; ERIC; SAGE Health sciences; ProQuest nursing and allied health source; OT Seeker; PEDro. A grey literature search was conducted of the following websites: The Australian Institute of Health and Welfare; Australian College of Rural and Remote Medicine; Australian Rural Health Education Network; Allied Health Professions Australia; CRANAPlus; Health Consumers of Rural and Remote Australia; Health Workforce Australia; National Rural Health Alliance; Rural Health Workforce Australia; Services for Australian Rural and Remote Allied Health. See Appendix for the full search terms as used in the Medline search. These terms were adjusted as necessary to suit each database searched.

For the purpose of this review, AHP were defined as speech and language therapists, nutritionists, dieticians, occupational therapists, physiotherapists, physiotherapy assistants, pharmacist aides, social workers or psychologists. This list of AHP was gleaned from the Australian Government Department of Health website [22]. Included articles must have over 50% AHP or must report results for AHP separately to other health professionals. Continuing medical education designed for physicians, doctors or nurses were excluded. We included any primary study designs (quantitative, qualitative and mixed methods) that offered distance education via lectures, workshops, seminars, symposia and courses by didactic or interactive means. The reference lists of opinion papers, commentaries and literature reviews were pearled for further relevant articles. Non-English language literature was excluded; no date restrictions were applied. EndNote software [23] and Covidence software [24] was utilised to manage the search results.

Each article was read for relevant data which was extracted into a customised data extraction table that was developed specifically for this systematic review. The extraction table contained key data domains, which were pertinent to the objectives and questions of this review including 1) study design; 2) sample size; 3) setting; 4) health discipline; 5) description of intervention; 6) technology used; 7) method of data collection; 8) outcomes reported and 9) results. The methodological quality of the included intervention studies was assessed using the Critical Appraisal Checklist for an Article on an Educational Intervention Tool [25].

The findings from individual studies were summarised depending on the types of evidence found for each question. Because the studies were heterogenous including qualitative, quantitative and mixed method designs, their findings were synthesised descriptively and emergent findings reported narratively [26]. All stages of the article selection and critical appraisal process were conducted by two independent reviewers; any discrepancies were resolved by discussion. A third independent reviewer made the final decision where discrepancies were not resolved.

### Search results

**Fig. 1:**
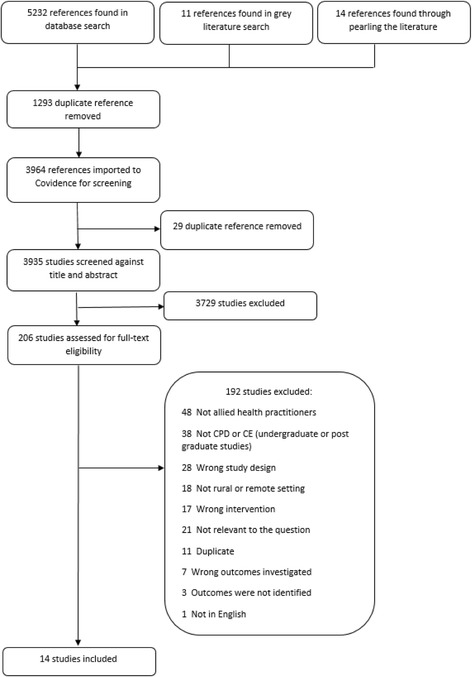
PRISMA flowchart of search and review process

The search of peer reviewed databases returned 5232 articles, a further 14 were found through reference list pearling and 11 reports were found in the grey literature search. After duplicate references were removed, the title and abstracts of 3964 articles were scanned to identify potentially relevant papers of which 206 full text were retrieved for a more detailed examination, and to ensure they met the inclusion criteria. Removal of duplicate publications and those that failed to meet the inclusion criteria resulted in 14 studies being included in this review (see Fig. 1 for PRISMA flow chart).

The 14 included papers were assessed for methodological quality of the study design [25].

The 14 included papers were assessed for methodological quality of the study design [25] (see Table 1 for details).

**Table 1:** Risk of bias appraisal of included papers

.		Bailey et al. 2005 [30]	Bynum et al. 2010 [31]	Dennis et al. 2010 [32]	DuBose et al. 1997 [27]	Ducat et al. 2014 [20]	Evans & Sachs 2000 [28]	Fahey et al. 2003 [38]	Maloney et al. 2011 [33]	Miller et al. 2008 [39]	Nipp et al. 2014 [35]	Ray et al. 2014 [36]	Shade & Barber 2004 [37]	Steed 2008 [29]	Warugaba et al. 2016 [34]
1	Is there a clearly focused question?	−	+	?	+	+	+	+	+	+	+	+	+	+	−
2	Was there a clear learning need that the intervention addressed?	?	+	+	+	+	+	+	+	+	+	+	+	+	+
3	Was there a clear description of the educational context for the intervention?	−	+	+	+	+	−	+	+	+	+	+	+	+	+
4	Was the precise nature of the intervention clear?	−	+	+	+	−	−	+	+	+	+	+	+	+	+
5	Was the study design chosen able to address the aims of the study?	?	+	?	−	+	+	+	+	+	+	+	+	?	?
6	Were the outcomes chosen to evaluate the intervention appropriate?	?	+	−	+	+	+	+	+	+	−	?	?	+	+
7	Were any other explanations of the results explored by the authors?	−	+	−	+	+	−	−	+	+	−	+	+	−	+
8	Were any unanticipated outcomes explained?	−	+	+	+	+	−	+	+	+	−	+	+	+	−
9	Reported behavioural changes after the intervention linked to measurement of other, more objective measures	−	?	−	−	−	−	+	+	−	+	?	−	−	−
10	Were the results of the intervention clear?*	+	+	+	+	+	+	+	+	+	+	−	+	?	+
11	How precise were the results?	?	+	−	+	+	+	−	+	+	+	−	?	−	−
12	Was the setting sufficiently similar to you own and/or representative of real life?	+	+	+	+	+	+	+	+	+	+	+	+	?	−
13	Does it require additional resources to adopt the intervention?	?	+	−	−	−	−	−	−	−	−	−	−	−	−
	*Risk of bias score out of 13*	*2*	*12*	*6*	*10*	*10*	*7*	*10*	*12*	*11*	*9*	*8*	*9*	*6*	*6*

Table key: + low risk of bias;? unclear; − high risk of bias; *question reworded for ease of dichotomous scoring (original question: ‘What were the results of the intervention?’)

### Findings

#### Study characteristics

The 14 included studies were published over a 19-year period from 1997 to 2016 (see Table 2). There were multiple studies conducted in Canada, Australia and the USA, while one focused on the needs of rural AHP in Rwanda. All but three studies [27–29] offered education to multi-disciplinary groups. CPD offerings varied and were either needs based, typically within health services or across health networks where topics were identified via surveys [30–32] or via topics selected by the University hosting the course [27]. Studies published prior to 2010 devoted large sections of their paper to describing the technology used for delivery of the education programs compared to more recent papers, which tend to discuss learning outcomes or pedagogies in more detail. All of the interventions were considered resource intensive and would require expense to establish and replicate. The most resource intensive intervention appeared to be Maloney et al. [33] who offered face to face sessions and compared learning outcomes with online delivery. They gave telephone support in addition to web based tutoring and access to the university technology support helpline 12 h a day, five days per week. Also resource intensive was Warugaba et al. [34] who collaborated with a university course design team to convert Massive Online Open Courses (MOOC) resources back into more basic technologies such as USBs and videos that were hand delivered to remote locations. Due to the heterogeneity of the studies, it was not possible to complete a meta-analysis of results and data were synthesised in a narrative form, with descriptive statistics (mean, standard deviation, standard error, number of students before and after intervention, effect size, *p*-value, t-value) reported where available (refer to Table 2 for the included study characteristics).

**Table 2:** Characteristics of the studies

Author, year and country	Design and data collection	Study purpose & participants	CPD topic	Outcome measures	Results
Bailey et al. 2005 [30] Australia	Service review (audit) Data collection method NR	AHPs Evaluate VC as a learning method Rural	Child development	Knowledge and clinical process	VC improved access to professional supports from metropolitan team; networking; knowledge in developmental disability and learning difficulties; enhancement of clinical processes
Bynum et al. 2010 [31] USA	Single arm post-test Self-report Likert scale	Total 44,989 with 3230 AHPs Evaluate satisfaction with education program using VC Rural	Varied, needs driven	Satisfaction with program length, presentation, effectiveness & convenience of technology. Satisfaction with impact on patient care	Rural participants reported highest satisfaction with technology convenience (*p* < 0.01), predictors of program satisfaction were program year, male (*p* < 0.01), African American (*p* < 0.01), healthcare discipline (nursing), community size (smallest) and travel mileage from originating site. Women (*p* < 0.01), Hispanics (*p* < 0.01) and dental professionals (*p* < 0.01) recorded greater increases in knowledge, and needs match. Multiple regression showed combined variables of program year, gender, ethnicity, healthcare discipline, home community size, and travel mileage to training site were significant predictors of program satisfaction, accounting for 5% of the variance (R^2^ = 0.05, *p* < 0.01). The strongest single predictor of satisfaction was program year
Dennis et al. 2010 [32] USA	Longitudinal cohort Self-reported pre-and post-Likert scale and end-of-year reflections	132 AHPs Evaluate learning from VC structured discussions Rural	Needs based journal club - critical appraisal	Critical appraisal skills; access to research and implementation	Access to research pre-to post 2006 to 2009 change of 3.10 to 3.88; critical appraisal skills change of 2.80 to 3.76; implementation change of 3.09 to 3.98
DuBose et al. 1997 [27] USA	Cross sectional cohort Examination and satisfaction evaluation (5 point Likert scale)	31 medical sonographers Evaluate education program comparing VC and FTF Rural and metro	Sonographic anatomy	Knowledge by rural versus classroom; overall; level of experience of participants & satisfaction	Students in remote sites did as well as those in classroom (*p* > 0.05), more years of experience had a small significant effect (*p* < 0.05, R^2^ = 0.42); satisfaction evaluation was generally good (mean 3.7, range 4.9 to 2.7). However, significant difference in satisfaction between rural and classroom (*p* < 0.05) with rural indicating feelings of isolation from other students and instructor.
Ducat et al. 2014 [20] Australia	Descriptive Qualitative Semi-structured interviews	42 AHPs Evaluate education program using blended delivery (TC, VC, FTF) Rural and remote	8 domains in line with the Allied Health Capability framework	Enablers and barriers	Barriers: Competing time demands; clinical work takes precedence; difficulty accessing the equipment for VC participation. Enablers: Access to VC was cost effective; no need for travel; efficiencies with staff time.
Evans & Sachs 2000 [28] USA	Cross sectional cohort Pre-and post-knowledge assessment with follow-up survey	378 sonographers Evaluate a TC with an expert panel Rural and urban	Ultrasound equipment developments	Satisfaction; relevance; knowledge	Overall satisfaction mean 4.5 (SD 0.60); relevance mean 4.55 (SD 0.61); between groups (managers and radiologic technologists) difference in satisfaction (*p* = 0.02) and relevance (*p* = 0.01); no gender differences in satisfaction *p* = 0.72 or relevance *p* = 0.94; satisfaction and relevance were correlated *p* < 0.001; knowledge scores improved from 85% to 95%
Fahey et al. 2003 [38] Australia	Cross sectional cohort Post session evaluation, surveys and interviews	38 AHPs Evaluate 12 session VC program Rural	Child psychological development	Knowledge; changes to practice; satisfaction with technology	Questionnaires: 80% felt the sessions were informative and self-report practice change would occur; 86% comfortable with technology; 12% discomfort; several stated ‘nothing replaces person in the room’. 80% rated online medium as excellent or very good, 1% unsatisfactory / poor. Acceptance consistently high from session 5 onward. Interviews (*n* = 16): 11 reported gains in knowledge in developmental frameworks and actual change in history taking & assessment; managers reported observed increased ability to spot problems; Networking was valued.
Maloney et al. 2011 [33] Australia	Head-to-head randomised trial Electronic survey for self-reported (Likert scale) satisfaction and self-reported change in practice, 1 h knowledge test, assignment	166 AHPs (attrition brought the final number to 96) Compare 1 day FTF workshop including video and written supports with web-based delivery over 4 weeks with discussion boards Rural and urban	Falls prevention using exercise	Participant reaction; knowledge; change in behaviour	Satisfaction content & relevance no difference (*p* = 0.75); satisfaction course facilitation & support no difference (*p* = 0.25); web group spent more time on compulsory & additional learning materials (*p* = 0.002); knowledge and assignment comparable between web and FTF (*p* = 0.07, *p* = 0.61); change in practice same (*p* = 0.89); difference in practice change between groups: web group changes in motivational interviewing while FTF changed exercise prescription. Both changed in assessment. Comfort with web based learning improved from 24% apprehensive to 80% willing to do another web based program.
Miller et al. 2008 [39] Canada	Non-equivalent control group design Self-reported pre-test and post-test and follow up survey for feedback using 5 point Likert scale, yes & no and open ended questions	44 AHPs Compare 1 day FTF workshop with VC delivered simultaneously Remote	Training in scoring guidelines for stroke assessment	Effectiveness acceptability & monetary costs	VC performed as well as FTF on pre-post-test of competency in scoring stroke assessment. Significant change in both groups between pre and post test scores *p* = 0.001) (i.e. learning occurred). 33% of FTF group thought training was excellent compared with 8% in VC group. Satisfaction in mode of participation was the same across both groups - the presence of the VC in the room did seem to impact the experience for the FTF attenders (i.e. reluctant to speak out as wanted to give VC chance to speak). VC was more cost effective
Nipp et al. 2014 [35] USA	quasi-experimental cohort Pre-and post-knowledge tests and follow up survey for practice change	28 AHPs Evaluate 5 continuing education modules delivered online Rural	Low vision assessment and treatment	Knowledge pre-and post-test; knowledge by years of practice experience	Change in knowledge was significant (*p* = 0.01). On follow up 73.7% indicated they consistently considered vision when planning treatment; 50% reported often screen for vision now and 15/19 participants now consider environment & vision. However, 63.2% did not use any of the screening assessments covered; 78% reported increase in comfort levels for providing interventions for low vision including increased activity visibility, increased contrast & organisation of work stations.
Ray et al. 2014 [36] Australia	Cross-sectional cohort Electronic survey using self-report Likert scales	Total 101, AHPs 20 Evaluate VCs with experts delivered monthly for 16 months Rural	16 Palliative care (PC) topics	Content usefulness, confidence of palliative care delivery & influence on practice	Content usefulness: significant difference in ratings between AHPs and MDs/students (*p* = 0.018) and nurses (*p* = 0.018); AHPs found content less useful than MDs and nurses. Practice location, years of working and number of clients seen were not significant. Confidence: AHP significantly lower confidence in topics than both nurses (*p* = 0.008) and MDs (*p* = 0.013); Overall confidence improved mean 0.54 (SD 0.46). Those who had more palliative care clients were more confident but years of experience had no effect. Change in confidence greater in those with no previous education than those with post-grad (*p* = 0.44) and short course experience (*p* = .014).
Shade & Barber 2004 [37] USA	Cohort Electronic survey after each course	58 AHPs Evaluate an adaptation of FTF education to online and video courses with peer support discussions Rural	Individualised gerontology instruction	Knowledge; satisfaction; ease of use; content, usefulness and application	Reported ‘average’ computer skills on program completion; high speed internet was an advantage. Not all course content translated easily to online environment; time consuming to design interactive experiences to compensate for no live facilitator; topics that were time-sensitive took effort to maintain but more static material was easier. Participants working together from a single agency enriched the learning experience and learner interaction.
Steed 2008 [29] USA	Mixed method case series Electronic survey after experience using Likert scale and open-ended questions	7 OTs Evaluate second life virtual reality as a learning method Rural and remote	Cultural competency	Attitudes about clients from a different culture perception of learning environment	4 themes: sense of presence - embodiment as an African American; Sense of co-presence - self in the environment with others; place presence - natural engagement supporting visual and kinaesthetic learning styles; sense of play - learning through fun - authentic and goal oriented.
Warugaba et al. 2016 [34] Rwanda	Cohort study Electronic survey after the education program	Total 38 completed: 17 were AHPs Evaluate an adaptation of a massive open online course including FTF support Rural and remote	Global health	Attendance at in-person classes; use of online forum, number of quizzes taken, time required, opinions whether course helps work and career advancement & learning	10 / 20 completers used online forums, 18 did up to 7 quizzes; 16 course was helpful to work, 18 course contributes to career advancement; 16 spend 2–5 h a week on course. Relationship between attendance at in-person classes and course completion statistically significant (*p* = 0.013).

Key: AHPs = Allied health practitioners; FTF = face to face; VC = videoconference; TC = teleconference; NR = not reported; MD = Medical Doctor

#### Critical appraisal

The two studies with the lowest risk of bias demonstrated thorough reporting of method and results but differed in two quality indicators; one reported the behavioural changes post educational intervention while the other provided enough detail for possible adoption of the method [31, 33]. The study with the lowest methodological score was a short report and unable to provide detail [30]. Overall the studies had a clear research question and adequately described the educational context and intervention although not with sufficient detail to enable replicability of the research. Most studies were evaluations thus it was difficult to control for variables in delivery of the intervention and context, making some of the studies quite complex and difficult to report concisely. The clarity of reporting of key results was consistent in most studies but some lacked precision of detail or the discussion of alternate explanations of results lacked deeper analysis which limited the usefulness of the research.

#### Outcome measures and methods of distance education

Primarily the studies evaluated domains of knowledge and satisfaction with learning processes or technologies used, while some also measured self-reported practice change. The main method of data collection was through course evaluations conducted by online or pen and paper surveys after the completion of the education. Some studies had both a course evaluation and a pre and post-test evaluation of self-reported knowledge change on a Likert type scale [28, 29, 32] and with open questions [33]. Some had an examination following the intervention [27, 33, 35], or formal assessment of knowledge before and after intervention [28]. Simpler study designs reported on findings from evaluation instruments administered only after delivery of the education [27, 29, 31, 36, 37]. In addition, three studies gathered qualitative data to evaluate learning and the utility of the methods of e-learning [20, 29, 38]. All evaluation instruments were bespoke, designed to ask about learning and specific aspects of the education that the participants did or did not find useful.

Those studies that tested knowledge found positive outcomes from the education programs regardless of method of delivery [28, 33, 35]. When video-conference was compared with face-to-face delivery of material there was a significant change in knowledge for both groups [33, 39]. However, those participating in a day long video-conference reported feeling fatigued, with sore eyes from looking at the screen [39].

There were only three studies [33, 35, 38] that clearly reported practice change following the educational intervention. Because they used self-reported measures of changes in knowledge or confidence, it cannot be assumed that there were resultant changes in practice. One study found that only half of their occupational therapy participants screened vision during assessments following education, meaning that whilst they were reported to be more confident, there was limited change in practice [35]. Nipp et al. [35] suggested that the limited change in practice could be due to the lack of interaction with facilitators and other students to promote learning. They recommended adding more interactive elements to e-learning courses to improve this outcome [35].

Some studies focused on evaluation of satisfaction with the CPD delivery method rather than the learning outcomes as their aim [20, 29, 30, 39]. Satisfaction with access, experience or usefulness of technology and subsequent learning processes varied across studies. Participants reported that they appreciated education that had an interactive component including contact with facilitators and other learners because it mirrored the kind of learning that occurs in the classroom and supported their engagement [29, 34, 36, 37]. The comparison of satisfaction with videoconference groups and face to face groups found no difference [39]. However, DuBose et al. [27] found there was a difference in satisfaction between rural participants and those in the classroom, with rural learners feeling isolated from the instructor and other students. In the Warugaba et al. [34] example, course completion was significantly related to attendance at the in-person classes that were an adapted addition to the original MOOC design. Multimedia delivery of content appeared to be favourable, possibly because this suited different learning styles [37]. The virtual reality experience in Steed [29] appeared to create immersion and a playful experience. However, the author indicated further data needed to be collected to determine if improvement in cultural sensitivity of participants occurred potentially highlighting the limitations of the education method.

Interactivity between learners supported networking between participants within rural areas or teams [37] and between rural and metropolitan participants [30]. However, interactivity was also cited as a negative indicator of satisfaction in some studies [27, 38], or a perceived constraint for verbal contribution when participants who were in the room with the facilitators felt the need to hold back to allow those who were at a distance to speak [39]. Shade and Barber [37] cautioned that designing interactive experiences that compensate for the absence of a live facilitator was time consuming.

The technologies used to deliver the distance education differed. Video-conference was a popular medium [20, 30–32, 36, 38, 39] including the oldest study in the review [27]. One study used relatively simple technology via teleconferences [28] with another creating a more complex intervention via a virtual reality situated learning experience [29]. Others used stand-alone or one topic offerings that were not administered through the internet such as videos [37] and narrated power-point presentations [35]. Other education was delivered through the internet using web-based systems that have multiple in built learning tools, such as Blackboard [37] or Moodle [33], to create online courses including asynchronous discussion boards [33, 37] and to offer MOOCs [34]. In a very remote region of Rwanda the online resources were not a suitable method of sole delivery and face to face supports were also provided [34].

Video-conferencing was found to be cost efficient in comparison to traveling to a larger city to attend a workshop [20, 39] and the relationship between distance and travel requirement was a finding in several studies. Bynum et al.’s [31] rural participants reported the highest satisfaction with technology convenience compared to videoconference users’ closer to the city from where the content was delivered. However, the duality of saved time, efficiencies and travel savings versus competing clinical demands and precedence when studying in situ was highlighted in Ducat et al.’s [20] analysis of the barriers and enablers of blended delivery methods.

Reduction in satisfaction was indicated by issues with readiness of learners to use the technology for education delivered via the internet or when they reported issues with bugs in the program, lag time, having to refresh the internet connection [29, 34, 37] and having limited access to the equipment needed [20]. This disruption affected the experience for learners and required patience for them to be supported in learning the technology as well as the content [29, 33]. Comfort with technology improved markedly in the Maloney [33] study from 24% *apprehensive* to 80% *willing to do another web based program*, suggesting offering support for technology use is a helpful addition to the suite of CPD options. However, actual satisfaction comparisons between the course facilitation and support in the face to face and web based offerings in the Maloney [33] study were not different.

## Discussion

Through conducting this review, once education targeted at medical practitioners was excluded, we found a surprisingly small range of research with variable methodological quality. This finding was surprising because of the many drivers for providing CPD for rural AHP including mandated professional registration requirements, the need to be professionally current [40], and to manage diverse practice demands [4]. Opportunities for engagement in CPD also minimises professional isolation, enhances quality improvement, and supports staff recruitment and retention [17]. In addition, CPD can inform rural AHP about product advancements and advancements in knowledge via implementation of research outcomes [41].

The research in this review was predominantly cross sectional with a mix of pre and post and quantitative measures and qualitative evaluations focusing primarily on knowledge development, satisfaction and utility with methods of education delivery and to a small degree on behaviour change and client outcomes. The almost 20-year span of the literature indicated that interest in effective modes of distance education is well established yet technology use is no longer novel. A pertinent observation was the trend of earlier publications to dwell extensively on descriptions of the technology, perhaps in an effort to enhance replicability, but to the detriment of the detail of the actual educational content or method. Both elements of the educational content and the method of delivery require attention to enhance replicability of the research. However, future efforts may benefit from giving more attention to the match between the method of delivery and the learning objectives of the program. For example, Evans and Sachs [28] demonstrated that for a straightforward session on new developments in a particular form of regularly used equipment, a low technology option of a teleconference could produce both knowledge gains and high satisfaction. Conversely, complex practice based courses may lend themselves more readily to either online or videoconference modes [33, 35, 36]. Similarly, education that requires a change in values and beliefs such as cultural sensitivity may require face-to-face contact for in-depth discussion [29], a finding congruent with studies of continuing medical education that indicate educational meetings alone are not effective for complex behaviour or practice changes [8].

Knowledge gains were a primary outcome of interest and all studies reported positive results regardless of the measures used, mode of technology, teaching and learning method, CPD topic or multi or sole disciplinary context. This finding suggests that AHP who opt to undertake CPD are likely to learn regardless, and perhaps the mode of delivery is not the most important aspect if knowledge alone is the desired outcome. The literature does not advance an understanding of the depth, longevity or application of that knowledge in practice despite efforts to measure practice change in two of the later Australian studies [33, 36].

Similarly, while it is suggested that provision of CPD is a strategy to retain staff [8], and while most studies measured satisfaction, none reported on retention as an outcome. It could be assumed that elements of the design of the different educational offerings may be of most benefit to retention of AHP in rural sectors. For example, studies with interactivity between participants and the facilitator appeared to have a higher satisfaction outcome, which is consistent with studies asking rural AHP about their CPD needs [10, 11] and evidence of strategies that produce the highest educational impact [8]. Therefore, it could be concluded that education that has an interactive element between the educator and the learner is better regarded by the recipient because they have the opportunity to discuss their learning. However, the nature of interactivity between participants was an intriguing finding of this review that deserves further research to determine which aspects of interactivity are most effective and how they may be facilitated via distance.

For example, networking opportunities through interactive means of education delivery were cited as beneficial [30, 38] but it appeared that if the education included videoconference participants off site as well as in class participants synchronously, those at a distance felt more isolated [27]. Presumably the goal of a CPD strategy for rural AHP is to reduce feelings of isolation rather than increase them therefore there is a need to carefully consider the best location mix of participants in each educational design.

With the exception of one paper, the research was conducted in USA, Canada or Australia, which are countries with vast distances between rural and metropolitan centres. The CPD strategies had similar purposes to reduce travel time and costs for rural AHP, which were established as achievable outcomes. However, the issue of travel is multifaceted; while reduced travel time was valued [20, 31], staying on site at the workplace to study could also be a barrier when competing clinical demands overtook time use [20]. Face to face versus distance modes was the focus of studies with comparative designs, testing the assumption that face to face learning produces better outcomes, which was not in fact supported when knowledge and satisfaction were both measured [33, 39].

A key new understanding from this review is the notion of the dynamic interaction between time use, travel, location, costs, interactivity, learning outcomes and educational design. On the surface, the results indicate distance education is well established and will produce good knowledge outcomes regardless of delivery mode. However, other aligned benefits such as professional networking opportunities, reducing rural isolation through building communities of practice virtually or in small local clusters deserve further consideration; the latter particularly when seeking to move from knowledge gains to changed practice behaviours and improved client outcomes. Further, simple modifications to design, such as potentially offering education via technology but in off work site locations away from clinical demands (e.g. in libraries or university rural departments) may reap additional benefits for recipients.

### Limitations and recommendations for future research

Given that existing literature supports the notion that access to CPD can aid in the retention of allied health practitioners in the rural workforce [13], it is a limitation of this review that no papers reported on retention as an outcome of engagement in distance CPD. An exploration of the relationship between availability of distance CPD and workforce retention is an area that is recommended for attention in future research. In addition, as this was secondary analysis of published research, we were not in a position to report on the motives of the participants for engagement with CPD and we did not know if participation was self-funded or employer funded. As evidence suggests that attendance is related to education outcomes, those who are most interested may also be those already performing well, and conversely those least interested may not attend and may need the CPD most [8] further research into the enablers and motivators for participation is important. Similarly, we were not aware of the motives of the CPD providers. This contextual information would be useful to interpretation and analysis of learning outcome data and is recommended for inclusion in reporting of future studies.

This study is limited by the lack of quality studies about distance CPD for rural allied health practitioners. In order to capture sufficient relevant studies, we had no date restriction on our search. This resulted in studies that spanned a 19 year period during a time of significant technological advancements. Comparisons were made about the different assumptions and emphases of authors from different periods of time, but comparisons between the different technologies need to made with caution and with understanding of the challenges that existed at those times (i.e. slower internet connections). It is recommended that future studies report detailed information about both the technology used and the educational intervention outcomes to advance understanding of the benefits and barriers to use of technology for distance delivery of CPD to rural allied health practitioners.

Schoo et al. [17] suggested that CPD for rural AHP should be based on core principles of professional group needs, adult learning principles and flexible delivery. The findings of this review suggest that these principles require deeper reflection, particularly the meaning of flexibility. Technology based delivery options appear to have high utility but perhaps flexibility and adult learning principles require more consideration above delivering distance education to a rural AHP desk top. Finally, while the studies showed some limited results, the extent and manner in which distance education CPD should be supported by additional knowledge translation strategies for change in practice, is of benefit to clients or improved service quality and whether it does in fact contribute to improve AHP retention in rural practice remains equivocal and is ripe for further prospective research.

## Conclusion

In this review, we have examined both published and grey literature describing the range of current distance learning strategies in use for providing CPD to rural AHP, in addition to evaluating effectiveness. The review has revealed a shift in focus from reporting on technology to reporting user satisfaction but evaluations of impact on practice are limited. Future studies could be enhanced by including detailed descriptions in order to enable replication, and further exploration of the complex relationships between instructional design, time use and location.

## Appendix

### Medline search

**Table 3:** Medline search. An academic librarian from the University of South Australia independently validated the search strategy

No#	Search terms	Results
1	((rural or remote or nonmetropolitan or non metropolitan or suburb*) and (health or health care or health servic*)).mp. [mp = title, abstract, original title, name of substance word, subject heading word, keyword heading word, protocol supplementary concept word, rare disease supplementary concept word, unique identifier]	89,254
2	*Rural Health Services/ or Rural Health/ or Suburban Health.mp. [mp = title, abstract, original title, name of substance word, subject heading word, keyword heading word, protocol supplementary concept word, rare disease supplementary concept word, unique identifier]	29,743
3	1 or 2	89,254
4	(((education or training) and (program or intervention or meeting or session or strategies or workshop or lecture or symposium or course)) or ((education or training) and (distance or remote or online or e-learning)) or Continuing professional education or Continuing professional development or CPD or CPE).mp. [mp = title, abstract, original title, name of substance word, subject heading word, keyword heading word, protocol supplementary concept word, rare disease supplementary concept word, unique identifier]	249,428
5	*education, continuing/ or education, pharmacy, continuing/ or education, professional, retraining/	5287
6	4 or 5	252,704
7	((allied health and (personnel or professional)) or occupational therap* or ((physical or occupational) and (therap* or assista*)) or physical therap* or physiotherapist or (speech and (therap* or patholog*)) or dietitian or dietician or diet* technician or pharmacist or (pharmacy and (technologist or technician))).mp. [mp = title, abstract, original title, name of substance word, subject heading word, keyword heading word, protocol supplementary concept word, rare disease supplementary concept word, unique identifier]	177,831
8	*allied health personnel/ or nutritionists/ or pharmacists’ aides/ or physical therapist assistants/ or physical therapists/ or Occupational Therapy/ or Pharmacists/	32,198
9	7 or 8	186,116
10	6 and 9	16,212
11	3 and 10	497

*Truncation symbol for boolean search

## Abbreviations

AHP: Allied Health Practitioner(s)
CPD: Continuing Professional Development
MOOC: Massive Online Open Course(s)
NDIS: National Disability Insurance Scheme
PRISMA: Preferred Reporting Items for Systematic Reviews and Meta-Analyses
